# Multiscale Ordinal-Pattern Dynamics and Temporal Symmetries in a Photonic Neuron with Single and Dual Delayed Feedback

**DOI:** 10.3390/e28050538

**Published:** 2026-05-09

**Authors:** Julian Feiveson, Mateu Yearian, Maddie Jones, Andrés Aragoneses

**Affiliations:** Physics Department, Whitman College, Walla Walla, WA 99362, USA

**Keywords:** complex dynamics, photonic neuron, chaos, multiscale dynamics, emergence

## Abstract

Feedback delays and the coexistence of multiple timescales are central features of complex dynamical systems, ranging from neural networks and ecosystems to electronic and optical devices. Interactions between fast and slow dynamics can give rise to rich emergent behaviors that are absent in single-timescale systems. Here we investigate how these coupled timescales shape the dynamics of a photonic neuron with single and dual delayed feedback. Using ordinal pattern analysis and recent ordinal-based complexity measures, we characterize the temporal correlations and symmetry properties of the fast peaks and slow spikes generated by the system. Our results show that the signatures of determinism exhibited at fast and slow timescales differ markedly, revealing a strongly multiscale organization of the dynamics. Despite these differences, when represented in the symmetry-based Φ-space, all cases, fast peaks and slow spikes under both single and dual feedback, collapse onto a common curve. This universal structure indicates the presence of underlying constraints governing the system’s dynamics across temporal scales and feedback configurations. These results highlight the power of ordinal-based approaches to uncover hidden symmetries and multiscale organization in delayed nonlinear systems.

## 1. Introduction

The interplay between coexisting timescales, usually induced by delayed feedback, provides natural mechanisms for memory, multistability, emergence, and complex temporal organization in complex dynamical systems, and has been studied from biological regulation and neural processing to lasers, electronic circuits, or mechanical oscillators. Despite this broad relevance, characterizing how dynamical structure and determinism emerge across coexisting temporal scales remains an open challenge, particularly in systems where fast and slow processes are strongly coupled. Developing frameworks to capture such multiscale organization is therefore essential to better understand delayed complex systems.

One of the defining characteristics of complex systems is time-delayed feedback. This, combined with disorder, diversity, and numerosity, gives rise to signature features such as emergence, self-organization, memory, and adaptive behavior [1,2,3]. Understanding and characterizing the role of feedback in complex dynamics is therefore essential for explaining and controlling such systems [4,5]. Another distinctive aspect of complex dynamics is its multidimensionality, in which interactions across multiple scales and variables shape the nonlinear evolution of the system [6,7,8,9].

These features naturally lead to the rich and often unpredictable behaviors observed in nonlinear and chaotic systems. A particularly compelling platform for investigating such dynamics is the photonic neuron [10,11,12,13,14,15,16,17,18], in which optical feedback enables fast and slow temporal scales to coexist, giving rise to complex emergent dynamics [19,20]. The interplay between these scales gives rise to complex firing patterns, memory effects, and emergent behaviors that cannot be captured by single-scale analyses. Introducing multiple feedback sources further enriches the system’s dynamics, allowing the exploration of how interactions between different temporal scales shape the overall dynamics [21].

In this study, we examine a photonic neuron subject to single and dual feedback, focusing on how fast and slow temporal dynamics interact to produce complex dynamical behaviors. This multiscale analysis not only highlights the intrinsic complexity of photonic neurons but also provides a framework, using ordinal pattern-based measures, for understanding and controlling their dynamics in neuromorphic photonic systems.

Photonic neurons are optical systems that can mimic the information-processing capabilities of biological neurons, enabling ultrafast, low-energy computation in neuromorphic photonic platforms [22,23,24]. A variety of architectures have been implemented, ranging from excitable semiconductor lasers and microcavity devices to integrated photonic circuits, each exploiting optical nonlinearities and feedback to generate neuron-like spiking and pulsing behavior [25,26]. These systems can implement fundamental neuronal functions such as excitability, thresholding, integration, and refractory dynamics, while operating on timescale orders of magnitude faster than biological neurons. By exploiting the inherent speed and flexibility of photonics, these devices provide a versatile testbed for exploring complex dynamics, multiscale interactions, and emergent behavior.

Among these architectures, excitable photonic neurons with delayed optical feedback provide a particularly rich platform for studying complex temporal dynamics [25,27]. The interplay between light-matter interactions inside the laser cavity and time-delayed feedback from a mirror introduces multiple coexisting timescales, giving rise to interactions between fast optical pulses (peaks) and slower envelope dynamics (spikes). This can generate emergent patterns, memory effects, and nonlinear temporal correlations. Introducing a second feedback can modify the temporal correlations and underlying complex dynamics, providing a way to probe how interactions between fast and slow timescales shape the system’s multiscale behavior.

## 2. Experimental Setup

**Figure 1:**
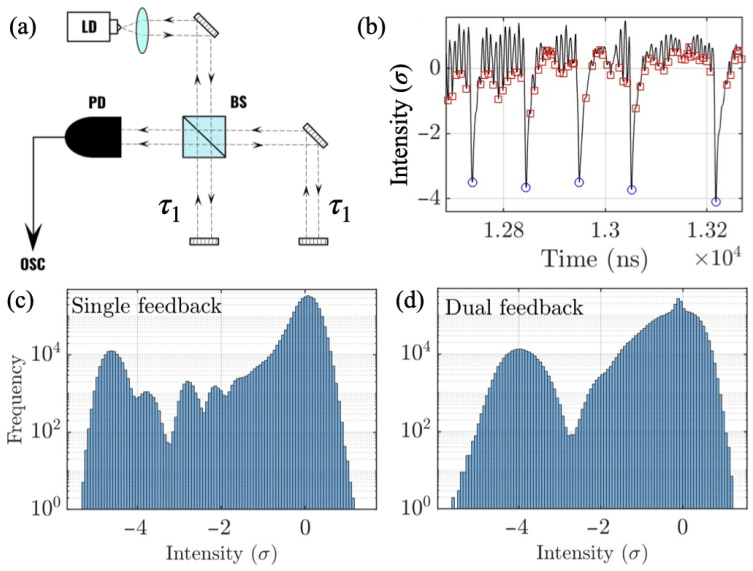
(**a**) Experimental setup. Light from the laser is sent to a 50:50 beam splitter (BS) and reflected off of two mirrors. Feedback strength is controlled through two neutral density filters (NDF). A photodetector collects the light and sends the signal to a 1 GHz oscilloscope. (**b**) Example of time series of the photonic neuron with dual feedback for *I* = 46 mA. The fast-dynamics peaks are marked with red squares and the slow-dynamics spikes are marked with blue circles. (**c**) Histogram (in logarithmic scale) of the peak depths for the single feedback configuration for *I* = 46 mA. (**d**) Histogram (in logarithmic scale) of the spike depths for the dual feedback configuration for *I* = 46 mA.

Our photonic neuron consists of a 650 nm diode laser (Thorlabs L650P007) with optical feedback implemented via one and two external mirrors (see Figure 1a). The feedback introduces time delays ($\tau_{1}=3.2$ ns and $\tau_{2}=4.5$ ns) that generate coexisting fast and slow temporal dynamics. When the ratio of time delays is an integer fraction there is a resonant effect that reduces the number of spikes considerably [28]. Our combination of time delays avoids that situation and improves the statistics.

When light from an external mirror is fed back to the laser its threshold is reduced from $I_{sol}$ to $I_{fb}$. Around these two currents new complex dynamics appear at the output power of the laser. The mount of feedback is quantified as the threshold reduction as $\eta =\frac{I_{sol}-I_{fb}}{I_{sol}}$. When 6%≲η≲12% the dynamics present fast shallow oscillations and slow deep dropouts [20]. Neutral density filters control the intensity of each feedback loop setting the threshold reductions to $\eta_{single}=10.0\%$ and $\eta_{dual}=8.5\%$, where the contribution of each mirror to the dual feedback was equivalent.

The laser’s pump current and temperature are stabilized to 0.01 mA and 0.01 C, respectively, using a Thorlabs ITC4001 controller. The output light is collected with a photodetector amplifier (Thorlabs APD210) and recorded with a 1 GHz oscilloscope (DSOS104A, Infiniium S-Series). Time series were acquired with a sampling rate of 2 Gsa/s, with a duration 2 ms per measurement.

This setup produces fast optical pulses (“peaks”) and slower envelope modulations (“spikes”), which serve as the fast and slow events for subsequent multiscale analysis. Figure 1b shows a portion of a time series where the fast peaks are indicated with red squares, and the slow spikes with blue circles. The time series are normalized to have zero mean and unit standard deviation σ. To identify the spikes we used a thresholding method where peaks below $I_{th}$ =−1.5σ are detected as spikes (see Figure 1b). For the fast peaks, their depth is −1.5σ<I<0.

Figure 1c,d show the histograms of the peaks detected for single and dual feedback configurations as a function of the detection depth in the time series. They clearly show different distributions, underlying the impact of an additional feedback source in the dynamical system. While the single feedback configuration shows a more intricate distribution of the peaks, with several regions (depths) of higher concentration of peaks, the dual feedback configuration shows a less complex, mainly bimodal distribution, where peak maxima are less concentrated and more spread out. These figures seem to indicate that a second feedback introduces robustness to the dynamics. As we will see in Section 4, the signatures of determinism for the dual feedback configuration are more pronounced.

## 3. Complexity Quantifiers

The dynamics exhibited by the photonic neuron present events at different temporal scales [19] as shown in Figure 1b. To investigate these multiscale interactions in detail, we employ ordinal-pattern analysis and recent complexity measures derived from ordinal sequences [29,30,31]. This approach allows us to quantify the temporal structure and signatures of determinism of the fast and slow dynamics, revealing how the interplay of peaks and spikes shapes the overall behavior of the photonic neuron.

Given a time series of events (peaks or spikes) we compute the time interval between consecutive events. By comparing *d* consecutive inter-event intervals we transform the time series of events into a time series of patterns of dimension *d*. For d=3, if $x_{i}<x_{i+1}<x_{i+2}$ we assign the pattern 012; if $x_{i}<x_{i+2}<x_{i+1}$ we assign the pattern 021, and so on. We obtain d! different patterns of dimension *d*. Our analysis is based on patterns of dimension d=3. We then calculate the probabilities of each of the 6 patterns, $P_{i}$.

While higher-dimensional patterns may capture more complex temporal correlations between consecutive events, d=3 already reveals correlations across both time scales that are incompatible with stochastic processes. Moreover, d=3 allows us to (i) visually inspect the evolution of ordinal pattern probabilities as the pump current varies, and (ii) define a geometric space in which reversible symmetries can be identified.

A visual complexity representation that captures temporal symmetries present in the dynamics is the ϕ-space introduced recently by Ansbacher et al. [31]. This three-dimensional space provides a geometrical representation where each point encodes the relative strength of time-reversible symmetries. This technique allows both dynamical complexity and symmetry structure to be visualized simultaneously. ϕ-space is defined as

(1) $$\left\{\begin{array}{c} \phi_{1}=\sqrt{-\frac{P_{1}\;ln\left(P_{1}\right)}{ln(6)}-\frac{P_{6}\;ln\left(P_{6}\right)}{ln(6)}}\\ \phi_{2}=\sqrt{-\frac{P_{2}\;ln\left(P_{2}\right)}{ln(6)}-\frac{P_{4}\;ln\left(P_{4}\right)}{ln(6)}}\\ \phi_{3}=\sqrt{-\frac{P_{3}\;ln\left(P_{3}\right)}{ln(6)}-\frac{P_{5}\;ln\left(P_{5}\right)}{ln(6)}} \end{array}\right.$$

where $\phi_{1}$, $\phi_{2}$, and $\phi_{3}$ are the three axes of this space. The probabilities $P_{i}$ are grouped into pairs corresponding to ordinal patterns related by time reversal ($P_{1}$ with $P_{6}$, …), such that each coordinate $\phi_{j}$ measures the contribution of a specific time-reversal symmetry class.

One interesting feature of this space is that $\phi_{1}^{2}+\phi_{2}^{2}+\phi_{3}^{2}=PE$, where PE is the Permutation Entropy introduced by Bandt and Pompe [29], where PE is computed as

(2) $$PE=-\frac{\sum_{i}P_{i}\;ln\left(P_{i}\right)}{ln(d!)}\;,$$

where 0≤PE≤1. A completely random process would lead to PE=1, while PE<1 would indicate the presence of some preferred patterns over others, revealing temporal correlations in the dynamics and signatures of memory.

Permutation Entropy is a global measure of dynamical complexity derived from the distribution of ordinal patterns, which encode temporal correlations among consecutive events. As a result, it is sensitive to the presence of memory in the underlying dynamics.

## 4. Results

**Figure 2:**
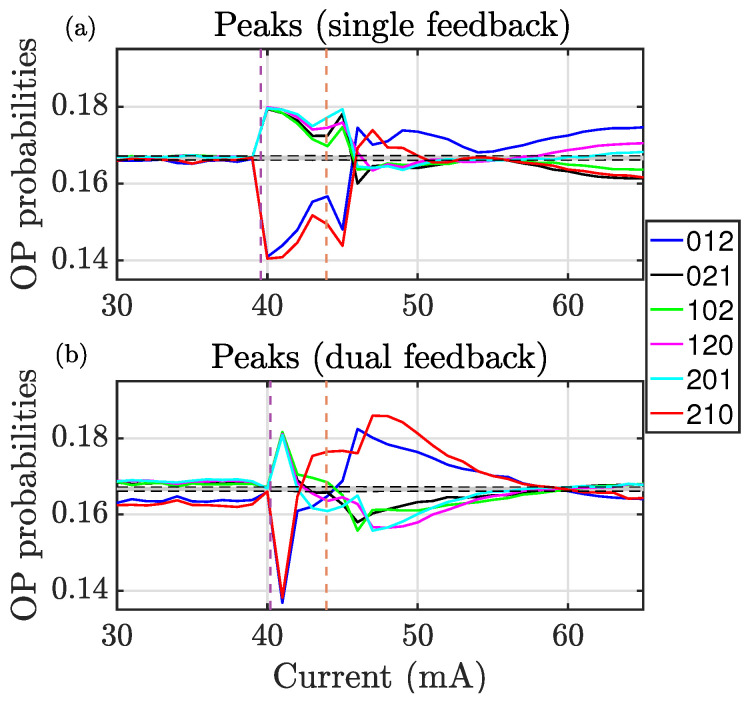
(**a**) Ordinal pattern populations (probabilities) for the photonic neuron with single feedback, computed with the time intervals between the peaks. (**b**) OP populations for the photonic neuron with dual feedback, computed with the time intervals of the peaks.

To quantify the temporal correlations at each time scale, we compute the probabilities of the six ordinal patterns of dimension d=3, computed with the time intervals between four consecutive peaks and consecutive spikes. Figure 2 shows these probabilities, which reveal the temporal correlations of the fast and slow dynamics for the photonic neuron with single feedback (a) and dual feedback (b). The vertical dashed lines indicate the laser threshold current with and without feedback. The central gray region corresponds to the null hypothesis of equally probable ordinal patterns, shown with a confidence level of 99.7% ($P_{x}\pm \sigma_{P}$, with $P_{x}=1/6$ and $\sigma_{P}=\sqrt{P_{x}\left(1-P_{x}\right)/N}$, where *N* is the total number of events in the time series). The number of events ranges from approximately 30,000 to 390,000 for spikes and is about an order of magnitude larger for peaks. As a result, the statistical uncertainties are very small and the error bars are not shown in the figures for visual clarity.

The range of pump currents spans from before the laser is on to when the dynamics enter into the more chaotic coherence collapse regime [32].

**Figure 3:**
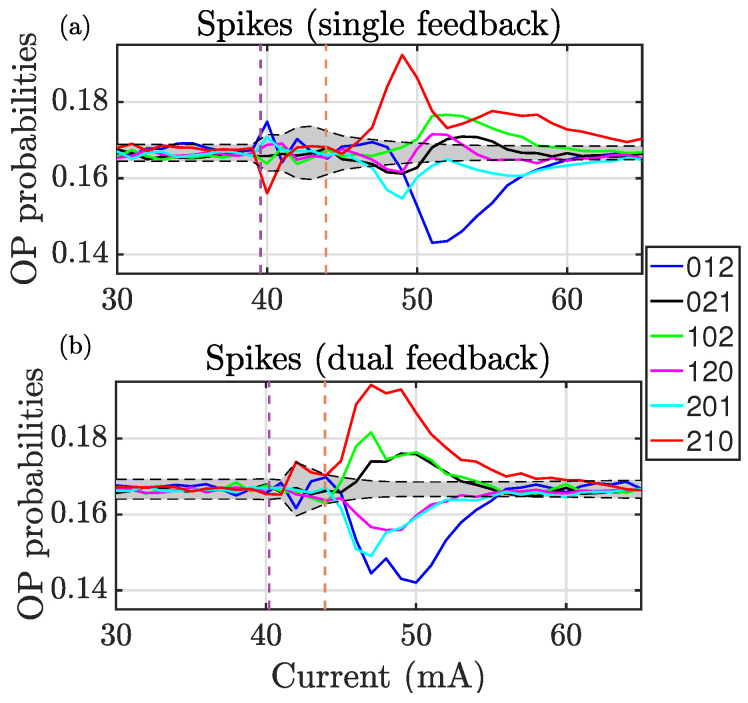
(**a**) OP populations for the photonic neuron with single feedback, computed with the time intervals between the spikes. (**b**) OP populations for the photonic neuron with dual feedback, computed with the time intervals between spikes. Vertical dashed lines indicate the threshold of the laser with (left) and without (right) feedback.

All four subplots in Figure 2 and Figure 3 exhibited both similarities and clear differences. As the pump current approaches the laser threshold (leftmost dashed line), a clear transition in the fast dynamics is observed for both single and dual feedback configurations. The ordinal pattern probabilities evolve from being compatible with a stochastic process to displaying statistically significant deviations from the uniform distribution, suggesting the emergence of deterministic temporal ordering.

Furthermore, a second transition in the fast dynamics is observed around the threshold current of the solitary laser (rightmost dashed line), where the ordinal pattern probabilities cross and reorganize into different hierarchies. Although the dynamics continue to exhibit signatures of determinism, the underlying temporal correlations differ from those observed at lower pump currents, indicating a qualitative change in the dynamical regime.

The slower spike dynamics also capture the transitions associated with the laser threshold currents (Figure 3). Near the lower threshold (laser with feedback), a broadening of the null-hypothesis region is observed, indicating an increased dispersion of spike events that persists up to the solitary laser threshold. In this interval, the ordinal pattern probabilities remain compatible with a stochastic process. Beyond the solitary laser threshold, the dynamics develop clear signatures of determinism, manifested as strong temporal correlations, with all ordinal pattern probabilities deviating significantly from the null hypothesis.

The hierarchy of the ordinal patterns in the slow dynamics (Figure 3), for both single and dual feedback, largely persists over the entire parameter range up to coherence collapse, where the probabilities gradually drift toward the null-hypothesis region, indicating a weakening of the temporal correlations.

A clear parallelism is observed between the single and dual feedback configurations in both the fast and slow dynamics. However, despite this correspondence, the hierarchies of the ordinal pattern probabilities in the fast and slow dynamics are qualitatively different, reflecting a multiscale behavior in which temporal correlations emerge and reorganize differently across time scales.

Interestingly, comparing the fast peak and slow spike dynamics between the two threshold currents reveals that the slow dynamics remain compatible with stochastic behavior, whereas the fast dynamics exhibit clear signatures of determinism. This demonstrates that, for these pump currents, temporal correlations are strong at short time scales but largely absent at longer ones. At higher pump currents, by contrast, temporal correlations emerge across all scales, though with qualitatively distinct signatures.

One notable feature revealed by these figures is that certain ordinal patterns form clusters whose probabilities evolve in tandem. Patterns 021 and 102 (or 120 and 201), which are related by a rotation symmetry [33], exhibit very similar probabilities. This clustering reflects the underlying temporal symmetries of the dynamics. While similar clustering has been observed at different time scales in the photonic neuron [19,20,21], it has not previously been explored in the fast-dynamics regime, where all peaks are considered without applying a detection threshold.

**Figure 4:**
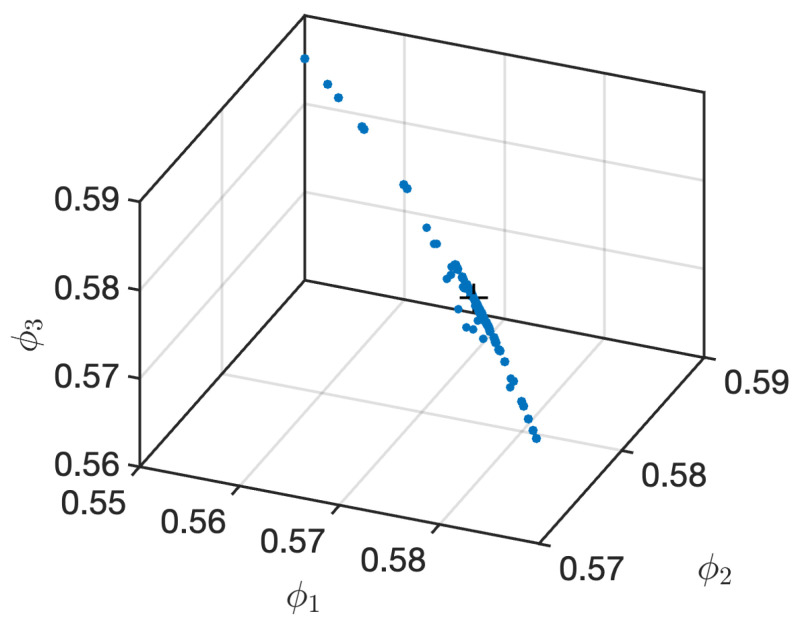
Φ-space visual representation for the ordinal patterns computed with the peaks and spikes for single and dual feedback. This reflects internal temporal symmetries present in the form clustering of groups of the populations of OPs.

A powerful visual tool for revealing hidden dynamical symmetries in a time series is the Φ-space representation. Figure 4 shows the projection of both the fast and slow dynamics for the single and dual feedback configurations across all pump currents in this three-dimensional space.

The location of all configurations and experimental conditions follows a well-defined three-dimensional curve, indicating that, although each combination of parameters is represented by a unique point in this space, they all satisfy underlying constraints that characterize the physical system. The clustering and geometric structures observed in Φ-space further suggest that the dynamics retain memory of previous events and are shaped by the interplay of the inherent dynamics of the laser and the feedback loops. The projection of all dynamics under study traces a clear curve, showing that despite the different behaviors unveiled across parameter space, a universal dynamical signature persists across all temporal scales.

Symmetries in the projections indicate recurrent temporal motifs, while deviations from perfect symmetry reflect how the system explores different dynamical regimes as the pump current varies.

## 5. Discussion and Conclusions

We have investigated the multiscale dynamics of a photonic neuron with single and dual delayed external optical feedback, focusing on the interplay between fast optical peaks and slower spike–envelope dynamics. By applying ordinal-pattern analysis and complexity measures we have characterized how temporal correlations emerge and evolve across different time scales as the pump current is varied.

Our results reveal that the fast and slow dynamics respond differently to changes in the operating conditions. In particular, near the laser threshold with feedback, the fast peak dynamics already exhibit clear signatures of determinism, while the slow spike dynamics remain largely compatible with stochastic behavior. As the pump current increases beyond the solitary laser threshold, temporal correlations emerge across both time scales, although with distinct ordinal hierarchies, highlighting the intrinsically multiscale nature of the system.

The comparison between single and dual feedback configurations shows that, while the overall qualitative behavior is preserved, the presence of multiple feedback loops enriches the dynamical structure and modifies the temporal correlations of both peaks and spikes. The clustering of ordinal patterns related by symmetry further indicates the presence of underlying dynamical constraints shaping the evolution of the system.

This is further captured by the Φ-space representation that provides an additional perspective and reveals that all experimental conditions collapse onto a well-defined curve in the symmetry-based complexity space. This result suggests the existence of universal dynamical constraints governing the photonic neuron, independent of the specific feedback configuration or temporal scale considered.

It will be interesting to explore in future research whether the geometric structures observed in Φ-space can be reproduced and explained within the deterministic Lang-Kobayashi model [10]. Such study could clarify the role of delayed times, feedback strengths, pump current, and noise in the dynamics.

While the present work analyzes two temporal scales separately, future studies could examine couplings between them, potentially revealing how fast optical fluctuations influence the slower envelope dynamics or vice versa.

Overall, this multiscale ordinal analysis provides a powerful framework for uncovering hidden temporal structures and symmetries in complex photonic systems. Beyond improving our understanding of the nonlinear dynamics of photonic neurons, these results may also contribute to the development of neuromorphic photonic architectures in which feedback and multiscale dynamics are harnessed for information processing and control.

## Data Availability

The datasets used and analyzed during the current study are available from the corresponding author on reasonable request.
